# Supplementary material for: A Metabonomic View on Wilms Tumor by High-Resolution Magic-Angle Spinning Nuclear Magnetic Resonance Spectroscopy
Source: Diagnostics (Basel). 2022 Jan 10;12(1):157. doi: 10.3390/diagnostics12010157 (PMC8775120; doi:10.3390/diagnostics12010157)
Supplement: Supplementary file 1 [file diagnostics-12-00157-s001.zip › diagnostics-1485569-supplementary.pdf]

Supplementary Material

A Metabonomic View on Wilms Tumor by High-Resolution Magic Angle Spinning Nuclear Magnetic Resonance Spectroscopy

Ljubica Tasic [1,*], Nataša Avramović [2], Melissa Quintero [1], Danijela Stanisic [1], Lucas G. Martins [1], Tassia B. Barroso Carneiro da Costa [1], Milka Jadranin [3], Maria Theresa de Souza Accioly [4], Paulo Faria [5], Beatriz de Camargo [6], Bruna M. de Sá Pereira [6] and Mariana Maschietto [7,†]

1 Laboratory of Chemical Biology, Institute of Chemistry, University of Campinas (UNICAMP), Campinas, Sao Paulo 13083-970, Brazil; meliquies@gmail.com (M.Q.); dacici.stanisic@gmail.com (D.S.); lgmartins1984@gmail.com (L.G.M.); tassiabrena@gmail.com (T.B.B.C.d.C.)

2 Faculty of Medicine, Institute of Medical Chemistry, University of Belgrade, Višegradska 26, 11000 Belgrade, Serbia; natasa.avramovic@med.bg.ac.rs

3 Institute of Chemistry, Technology and Metallurgy, Department of Chemistry, University of Belgrade, Njegoševa 12, 11000 Belgrade, Serbia; milkaj@chem.bg.ac.rs

4 National Bank of Tumors of National Cancer Institute (BNT-INCA), Rio de Janeiro 20231-091, Brazil; maccioly@inca.gov.br

5 Department of Pathology, Federal University of Rio de Janeiro (UFRJ), Rio de Janeiro 21941-901, Brazil; pauloafaria@gmail.com

6 Clinical Research Department, National Cancer Institute (INCA), Rio de Janeiro 20231-091, Brazil; bdecamar@terra.com.br (B.d.C.); pereira.brunams@gmail.com (B.M.d.S.P.)

7 National Laboratory of Biosciences (LNBio), National Center for Research in Energy and Materials (CNPEM), Campinas, Sao Paulo 13083-100, Brazil; marianamasc@gmail.com

* Correspondence: ljubica@unicamp.br

† Current address: Boldrini Children's Hospital, Campinas, Sao Paulo 13083-884, Brazil

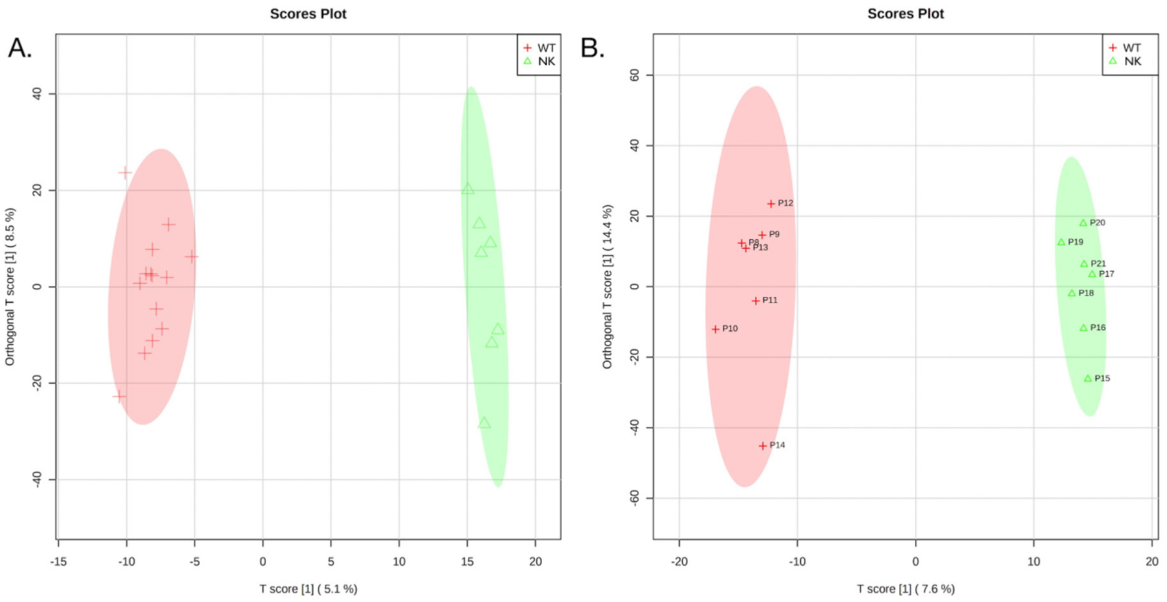

Figure S1. oPLS-DA scores plots in 2D, T score vs. Orthogonal T score, of the HR-MAS ^{1}H-NMR CPMG spectra models: (**A**) constructed using 21 tissue samples, 14 WT and 7 NK (Permutation analysis R^2Y: 0.878 for $p < 0.05$, and Q^2: 0.620 for $p < 0.05$); (**B**) constructed for 7 paired WT and NK samples (Permutation analysis R^2Y: 0.787 for $p < 0.05$, and Q^2: 0.677 for $p < 0.05$). WT tissue samples are shown in the red crosses and normal kidney (NK) tissue samples are shown in green triangles.

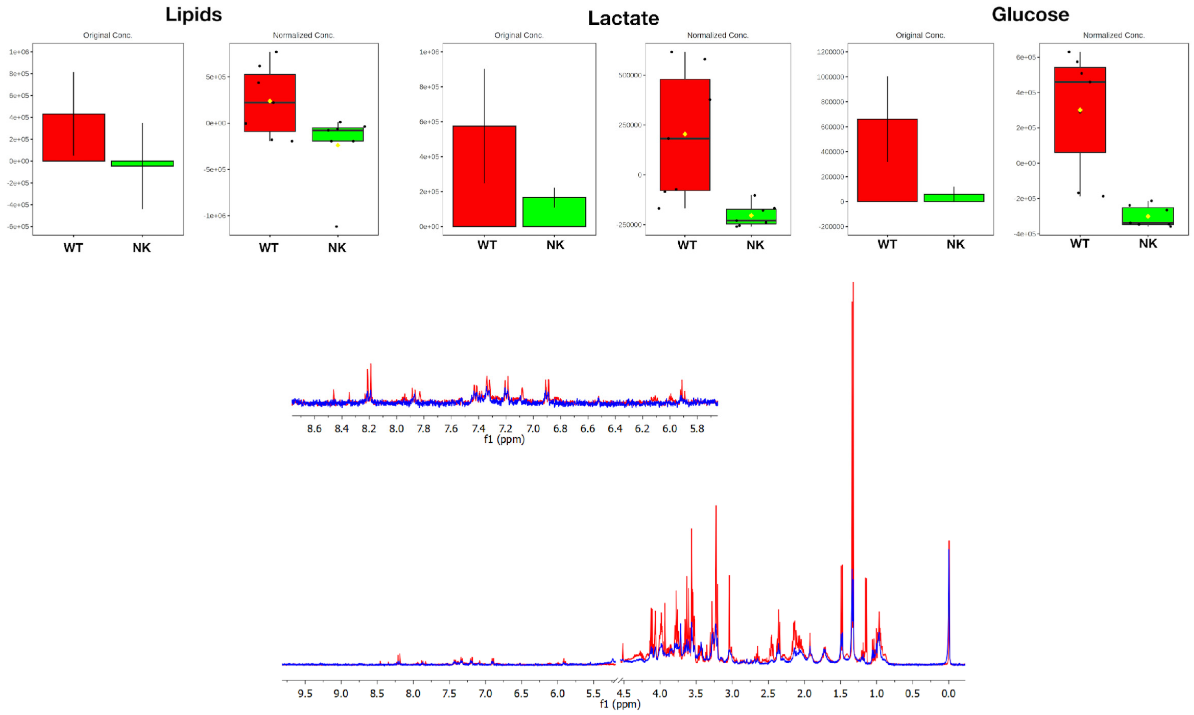

Figure S2. Box plots of Lipids (0.803 ppm), Lactate (1.33 ppm), and Glucose (metabolite 13, see Figure 1) variation according to t-test univariate analysis in paired cancer tissue samples, 7WT vs. 7 NK; WT tissue samples are shown in the red and normal kidney (NK) tissue samples are shown in green (upper panel). Superposition of ^{1}H-NMR (CPMG HRMAS) of WT (red) and NK samples, shown in the lower panel. Just 0.50–4.50 ppm regions were used for PLS-DA and oPLS-DA models.
